# Orthodontic camouflage treatment of pseudo‐Class III malocclusion on skeletal class III Base complicated with canine impaction treated with temporary anchorage devices: A case report

**DOI:** 10.1002/ccr3.7394

**Published:** 2023-06-07

**Authors:** Saud Alotaibi

**Affiliations:** ^1^ Department of Preventive Dental Sciences, College of Dentistry Majmaah University Al‐Majmaah Saudi Arabia

**Keywords:** class III malocclusion, impacted canine, orthodontic appliance designs, orthodontic camouflage, temporary anchorage devices

## Abstract

With conventional mechanics to protract the upper posterior teeth for correcting Class III molar relationships, such as intra‐arch mechanics, face mask reverse‐pull headgear protraction, and interarch Class III elastics, there are some adverse effects, including diminished patient compliance, the possibility of losing anchorage, and extrusion of upper molars and lower incisors with counterclockwise rotation of the occlusal plane. Protraction force should be directed through the center of resistance of the upper posterior teeth to prevent these side effects.

## 1 INTRODUCTION

Angle's classification of malocclusion was the first and most recognized way to classify dental malocclusion into four classes, including normal occlusion, Class I, Class II, and Class III malocclusion based on the interarch relationship of the maxillary and mandibular first molars and the alignment of the teeth concerning the line of occlusion.^1^ In addition, Angle's classification is used to diagnose the underlying skeletal jaw relationship of the maxilla and the mandible to each other, despite the molar relationship. Thus, a skeletal Class III relationship means that the mandible is positioned forward of the maxilla or is positioned in a retruded position relative to the mandible or both. This relationship could be found with a Class III molar relationship or occasionally a Class I molar relationship when there is an overcoming dental compensation. The prevalence of Class III malocclusion varies among populations and has a relatively low incidence with the global prevalence of 3.98%. Its prevalence is high in Asia with 6.46%, followed by Africa with 4.6%, and Europe and America with the lowest prevalence of 2.75% and 2.68%, respectively.^2^ Malocclusion classified as Pseudo‐Class III is commonly observed in primary and mixed dentition, with approximately 5.8% of anterior crossbites in 6 to 12‐year‐old patients.^3^ The anterior forward displacement of the mandible due to interferences producing anterior crossbite is a characteristic feature of Pseudo‐Class III malocclusion. An accurate diagnosis of Pseudo‐Class III malocclusion plays a crucial role in devising the orthodontic treatment plan. After guiding the patient into a centric relationship position, As the incisors show an edge‐to‐edge relationship with posterior open bite, and the posterior teeth occlude in centric occlusion, a forward functional mandibular shift is created, causing the incisors to occlude in an anterior crossbite, which confirms Pseudo‐Class III malocclusion.^4^ An accurate diagnosis must be obtained by distinguishing between true Class III malocclusion and Pseudo‐Class III malocclusion to reach a precise treatment plan. Treating adult patients with a related skeletal deformity has special considerations. For example, the patient's vertical growth pattern affects the prognosis of the treatment with poor prognosis observed in vertical growth tendency cases. If the patient has a severe skeletal discrepancy and the camouflage orthodontic treatment is not an option, orthognathic surgery will be the only approach to obtain both functional occlusion and esthetic objectives. Supposing the skeletal discrepancy is mild, there is room for dentoalveolar compensation without adversely affecting the teeth’ soft tissue profile or periodontal health. In that case, camouflage orthodontics alone could achieve a functional and stable occlusion without dramatic esthetic changes in the profile, which is an advantage of the orthodontic orthognathic approach.^5^ However, orthognathic surgery implicates some biological and economic factors that could affect the decision‐making during treatment planning of such cases. Camouflaging Class III malocclusion main objectives can be summarized into two mechanics. Distalization of the mandibular teeth to correct a Class III molar relationship, or protraction of the maxillary dentition with proclination of the upper anterior teeth to achieve a positive overjet. It can be achieved with two different mechanics: extractions (extraction of upper second premolars and lower first premolars, lower first premolars alone, or one lower central incisor depending on the case) or nonextraction mechanics. Lower molar distalization is a complicated movement to do in orthodontics.^6^ This goal has been attained using a variety of devices and approaches, including mandibular headgear, lip bumpers, distal extension lingual arches, Jones jigs, Franzulum appliances, multibrackets with Class III elastics, and edgewise treatment loops.^7^ On the contrary, protraction of the upper dental arch was limited by using Class III elastics or face mask reverse‐pull headgear. Because all these treatment mechanics depend on the opposing arch dentition for anchorage, they have some unwanted effects, like extrusion of teeth, increasing lower anterior face height, and counterclockwise rotation of the occlusal plane. Temporary anchorage devices (TADs) can provide an absolute anchorage to prevent these unwanted effects because they are easy to place and remove, low cost, and can be placed in multiple places. These TADs are helpful for various difficult tooth movements, like intrusion, retraction, and protraction.^8^

## 2 CASE REPORT

### 2.1 Case history and diagnosis

**FIGURE 1:**
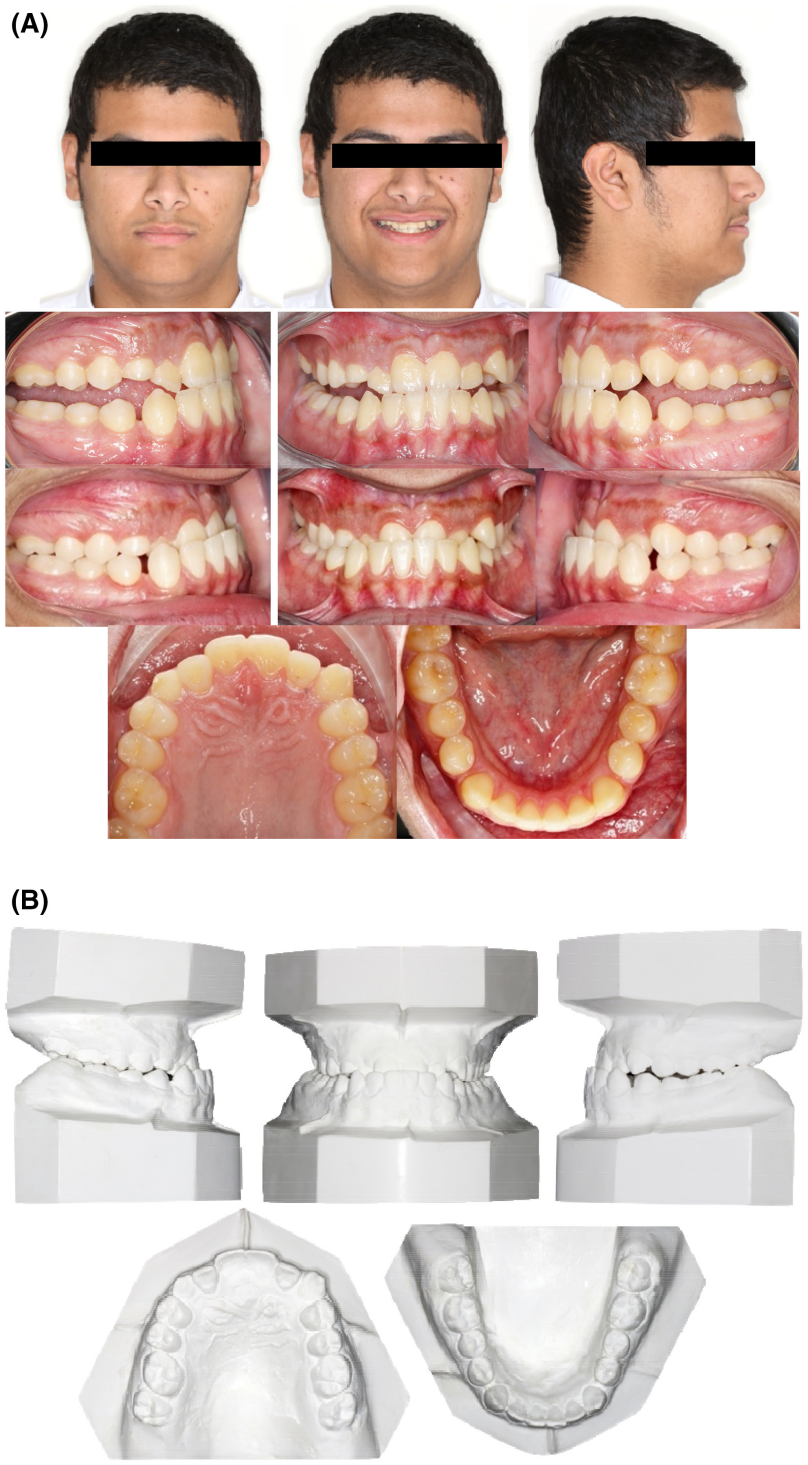
(A) Pretreatment extra and intraoral photographs in centric relation (CR) and centric occlusion (CO). (B) Pretreatment orthodontic casts.

A young male patient aged 20 years old, who is a college student is the subject of this case report. He was not aware of any medical problems or was using any medications. He brushes his teeth twice a day; last dental history was an examination by the general dentist and referral to the orthodontic department. His chief complaint is “I can't close my teeth well”. There was no habit reported, and the temporomandibular joint (TMJ) was normal. A mesofacial appearance with a straight profile is seen in (Figure 1A). He presented first with Class III molar and canine relationship, anterior crossbite, reduced maxillary intermolar width, retained #53, and impacted #13 (Figure 1B). Clinical evaluation revealed that there was a functional shift at closure. After centric relation‐centric occlusion (CR‐CO) correction of the cephalometric radiograph, the cephalometric tracing showed a mild skeletal Class III due to retrognathic maxilla, anteriorly inclined maxilla, deep basal configuration, and hypodivergent mandible (14.6°). The final diagnosis was Pseudo‐Class III malocclusion on mild Class III skeletal base due to retrognathic maxilla complicated with impacted upper canine and reduced intermolar width in the upper arch. Periodontal health was good, with fair to good oral hygiene. He breathed nasally and has a strong internal motivation for orthodontic treatment.

#### 2.1.1 In frontal extraoral examination (at rest)

He has a fairly symmetrical face with mesofacial form, competent lips, and average lower anterior facial height.

#### 2.1.2 In frontal extraoral examination (at smile)

He has a low smile line, upper dental midline coincides with the facial midline, 0 mm of gingival show upon smiling, no occlusal cant, and wide buccal corridor.

### 2.2 Radiographic analysis

#### 2.2.1 Panoramic radiograph

**FIGURE 2:**
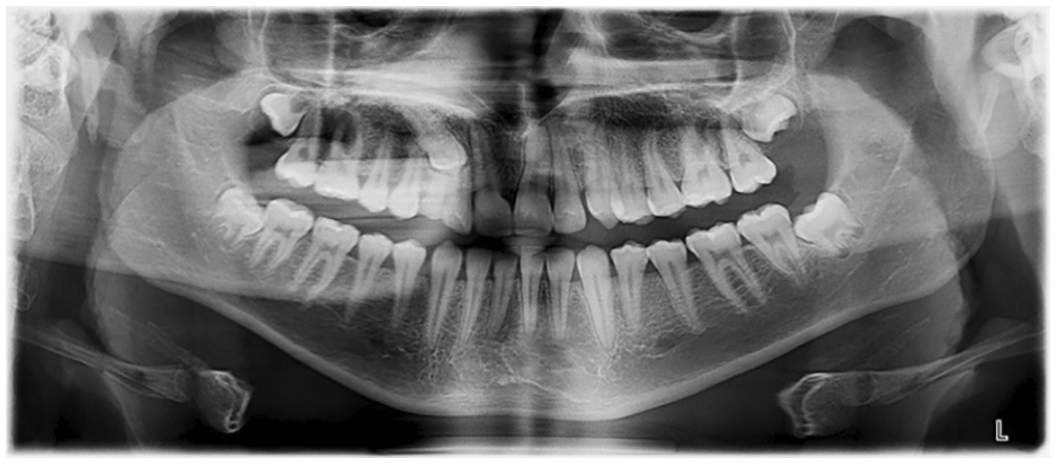
Pretreatment panoramic radiograph.

Revealed symmetrical condyles and normal ramus, the body of the mandible, and maxillary sinuses. All teeth were present, impacted #13, and retained #53. Third molars were erupting (Figure 2).

#### 2.2.2 Cone beam computed tomography (CBCT) Scans

**FIGURE 3:**
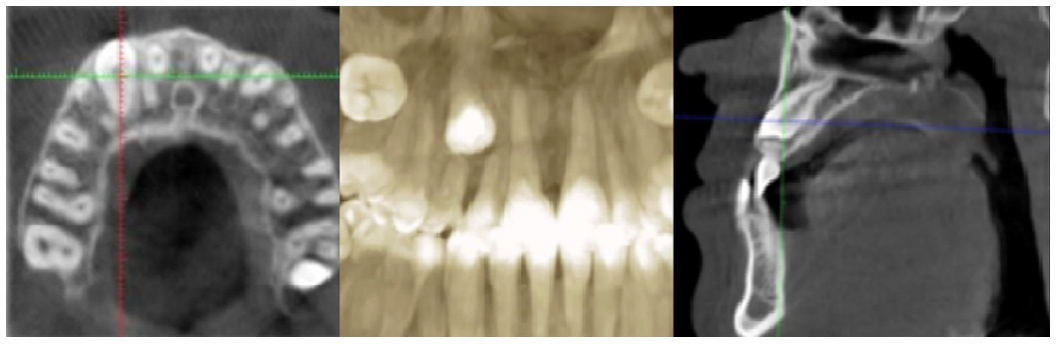
Pretreatment (CBCT) scans.

As seen in these scans (Figure 3), #13 was vertically impacted with a good prognosis and positioned on the labial side with no bone covering the crown labially. The canine was close to #12.

#### 2.2.3 Cephalometric radiograph

**FIGURE 4:**
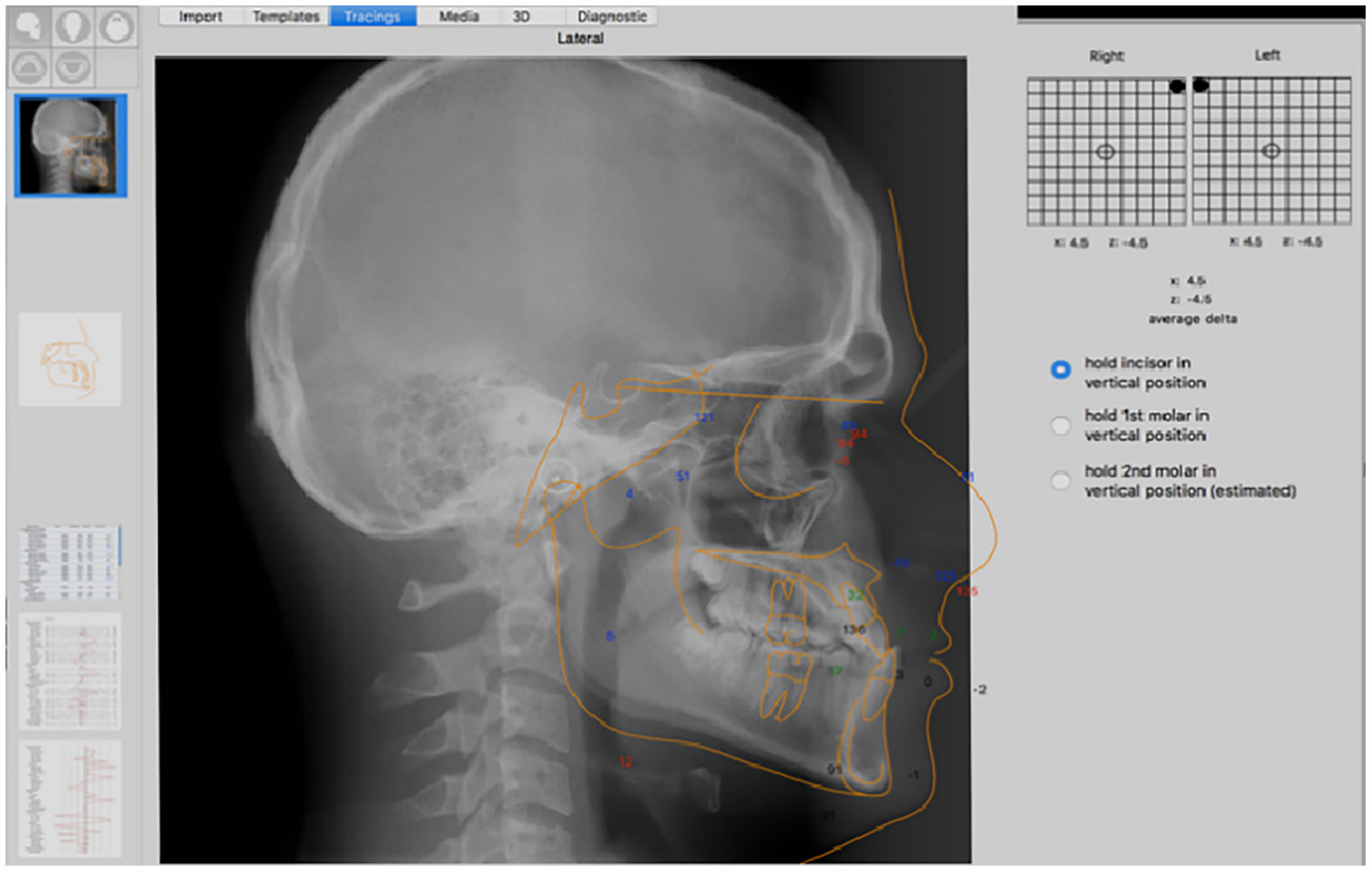
CO‐CR Conversion tool in "Quick ceph" studio software.

With the new advances in cephalometric tracing software, we can avoid exposing patients with functional shifts twice to have two radiographs in centric occlusion and centric relation positions. Moreover, by utilizing the CO‐CR Conversion tool in “Quick ceph” studio software (Figure 4), we can reposition the mandible from centric occlusion (CO) (habitual position of the mandible, where the radiograph has been taken) to centric relation (CR) (the seated hinge axis of the mandible).

**FIGURE 5:**
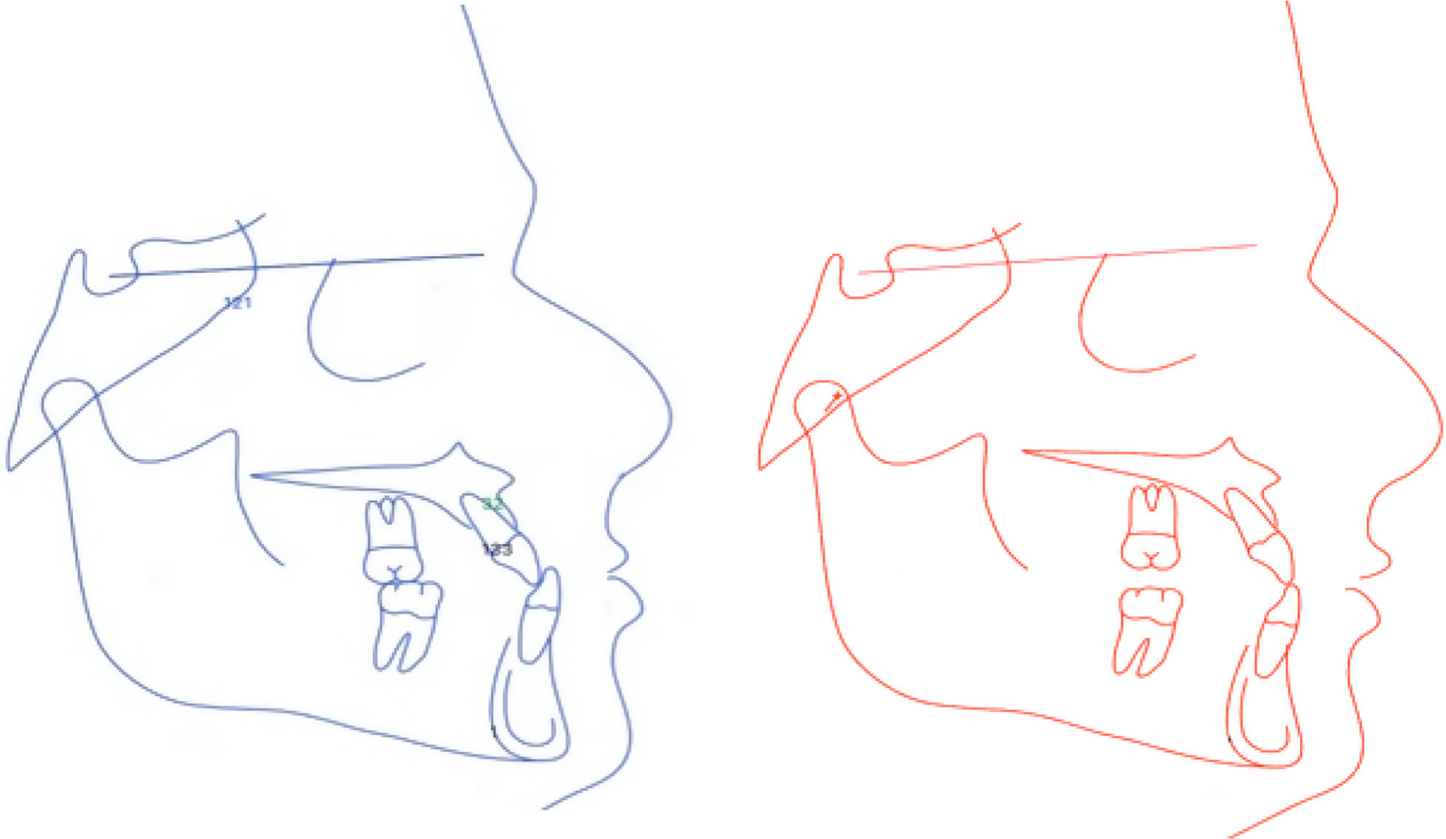
CO‐CR shift cephalometric correction.

Using a reference guide, such as the semiadjustable articulator with a facebow transfer, bite registration or clinical photographs, the mandible can be repositioned to the centric relation position. Then, the vertical measurements from the CO tracing and sagittal measurements from CR tracing can be obtained (Figure 5). The final cephalometric tracing results after the correction as observed in Table 1.

**TABLE 1:** Cephalometric measurements.

Measurement	Norms	Pre‐Tx	Post‐Tx
Sagittal (apical bases and chin)			
SNA	82° ± 2°	78°	77.8°
SNB	78° ± 2°	82°	82°
ANB	2° ± 2°	−4°	−4.2°
Wit's appraisal	‐1 mm/ 0 mm	−7 mm	−7.5
SN‐Pog	80° ± 3°	83.6°	83.5°
NA‐A Pog	0° ± 5°	−4°	−4.3°
Vertical/divergency			
SN‐MP	32° ± 5°	14.6°	16.5°
SN‐PP	8° ± 3°	4.4°	4.2°
PP‐MP	25° ± 3°	10.2°	12.3°
Me‐tgo‐Ar	126° ± 10°	109.8°	109°
Y‐axis	59.4° ± 3.8°	51°	53°
ANS‐Me/N‐Me	55 ± 3%	55.9%	56.5%
Dental (incisor position)			
U1‐L1	131° ± 5°	135°	137°
UI‐SN	104° ±2°	111°	118°
UI‐PP	110° ± 6°	125°	132°
UI‐NA	22° (4 mm)	32° (5 mm)	35° (8 mm)
LI‐NB	25° (4 mm)	26° (4.3 mm)	22° (3 mm)
LI‐Apog	1 mm ± 2 mm	4 mm	3.5 mm
L1‐MP	93° ± 6°	95.6°	89.5°
U1‐L1	131° ± 5°	135°	137°
Soft tissue			
UL‐EL	−4 mm ± 2 mm	−3.1 mm	−2 mm
LL‐EL	−2 mm ± 2 mm	−2.6 mm	−1.5 mm
NLA	90°‐110°	115°	111°

#### 2.2.4 Treatment objectives

As mentioned earlier, the final diagnosis summary was “Pseudo‐Class III malocclusion on mild Class III skeletal base due to retrognathic maxilla, complicated with impacted upper right canine and reduced intermolar width in the upper arch.” The ideal treatment plan for this case is the surgical approach, but he refused it and insisted on treating him without surgery. For nongrowing Class III skeletal malocclusion patients, the surgical option would provide the patient with excellent results and incredible changes in the soft tissue profile. The final treatment objectives were based on a camouflage orthodontic treatment that accepting the skeletal discrepancy by utilizing a nonsurgical, nonextraction comprehensive orthodontic treatment with palatal expansion to increase the arch perimeter and correct the intermolar width and exposure and retraction of the impacted canine.

### 2.3 Treatment progress

**FIGURE 6:**
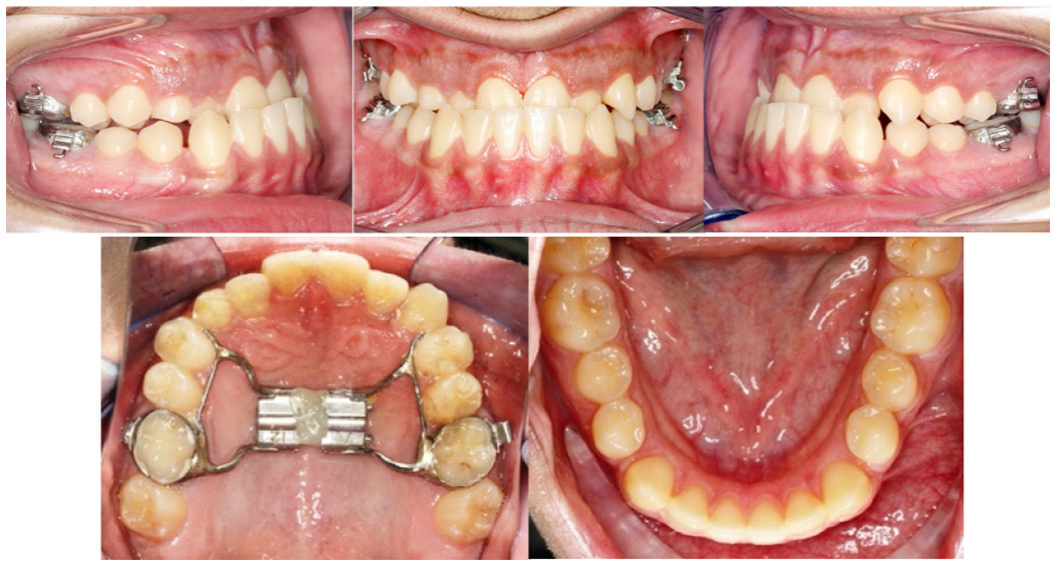
Stabilized hyrax expander with composite resin for 3 months.

The first step in the treatment was the expansion phase. Hyrax expander was used just to have some dental expansion, with instructions to the patient to do one turn every other day (slow expansion). After overcorrection is achieved, the expander be stabilized with composite material for 3 months (Figure 6).

**FIGURE 7:**
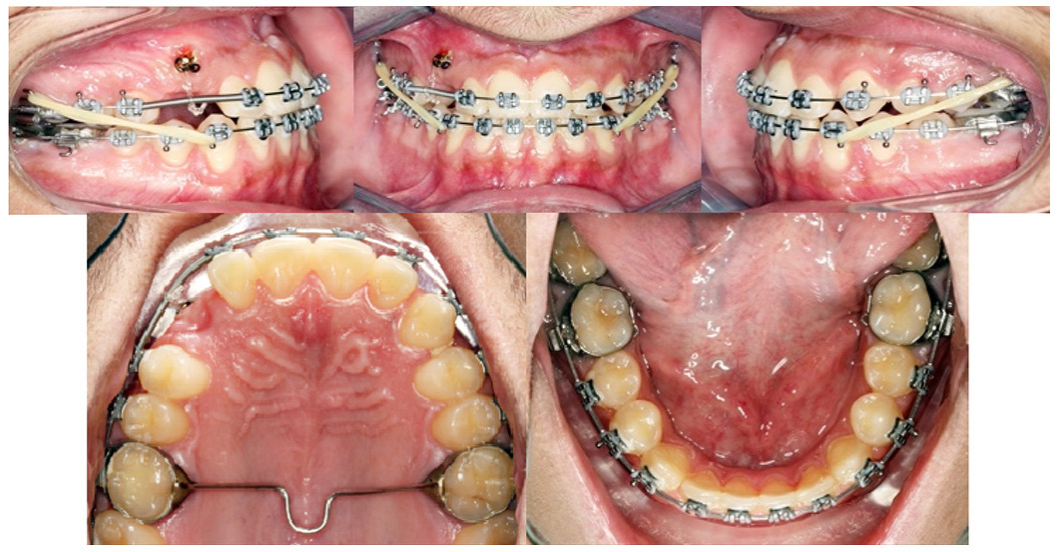
Bonded gold Bottom to the canine with the closed flap.

The expander was removed and the transpalatal arch (TPA) was cemented for more retention. Bonding upper second molars was done with bonding from 5 to 5 using 0.018 slots. MBT prescription brackets with passive bonding was done for #12. followed by leveling, alignment, and derotation using 0.014 “Nickel Titanium (NiTi) wire. The bite was raised by blue resin. The wire was progressed to 0.016” (NiTi) for three visits before upgrading to 0.016 × 0.022 “(NiTi). After reaching to upper archwire 0.016” X 0.022 “Niti, banding of the lower first and second molars and bonding the lower arch 5‐5 using 0.018 slot MBT prescription brackets. Leveling, alignment, and de‐rotation was started using 0.016” (NiTi) wire. Light Class III elastics (5/16 Light) was used and bite risers were continually used to help correction of the anterior interference. The 0.016 “(NiTi) wire was used for 2 visits before upgrading to 0.016x0.022” (NiTi). After that, upgrading to 0.016" X 0.022" stainless steel (SS) wires, then the removal of the bite risers was gradual with using Class III Elastics (5/16 Medium) full time. The patient was referred for extraction of #53, exposure of #13, and bonding of gold‐chain to the tooth with the closed flap method, followed by retracting the canine to the line of occlusion by an elastic thread (Figure 7).

**FIGURE 8:**
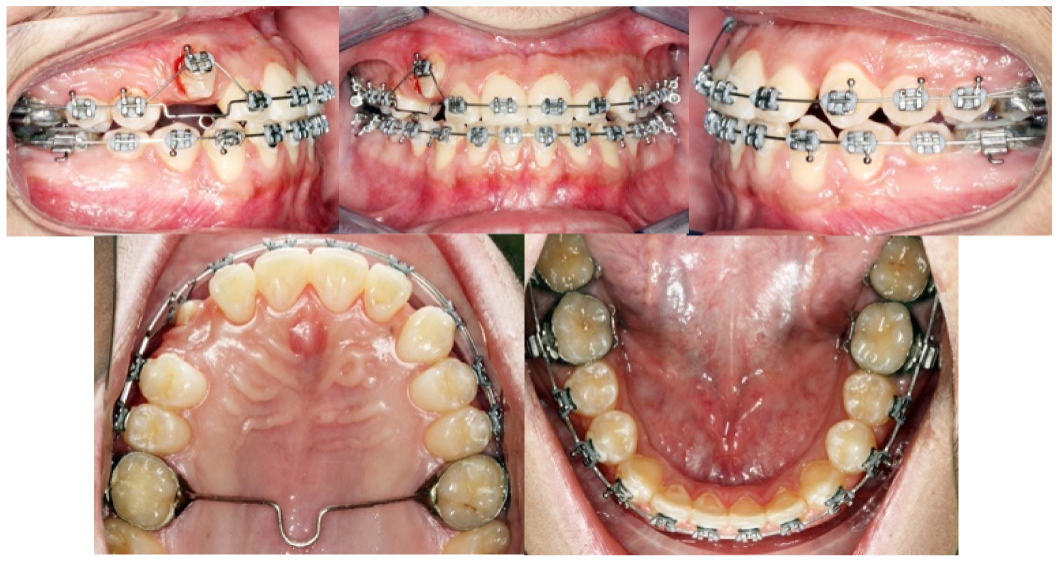
Stabilizing archwires and leveling with overlay 0.012” Niti wire.

After #13 was fully exposed and near the arch, a bracket was bonded and leveling was started using overlay 0.012" NiTi wire (Figure 8).

**FIGURE 9:**
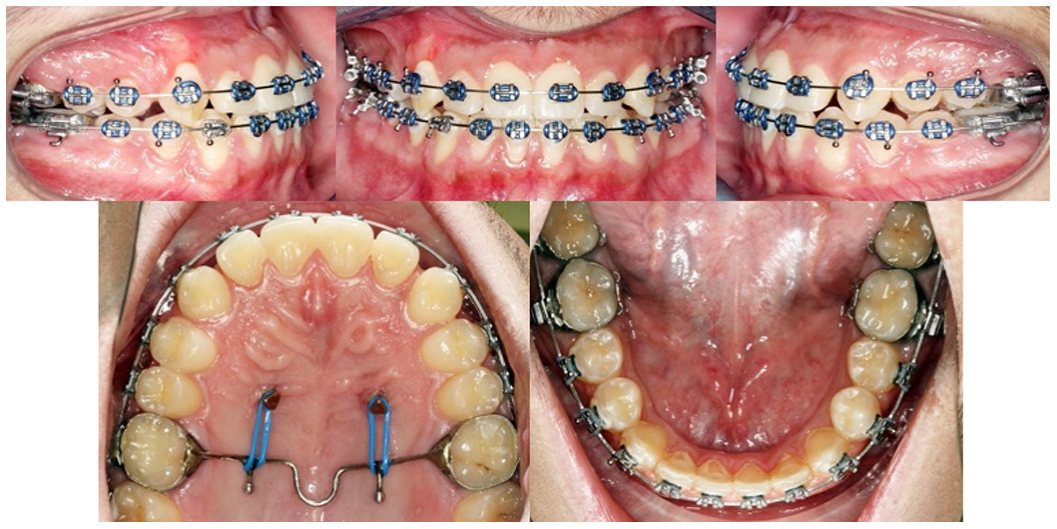
TADs anterior to the TPA to apply protraction movement through the center of resistance of the upper posterior teeth.

After the canine was already in place, two paramedian palatal TADs were placed (11 mm in length and 1.5 mm in diameter) anterior to the TPA with soldering hocks in the TPA apically near the center of resistance of the upper molars (Figure 9).

**FIGURE 10:**
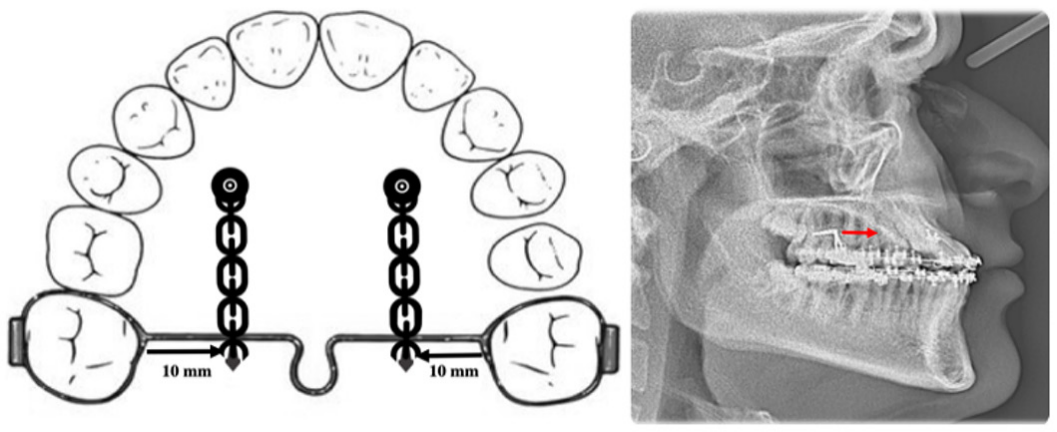
TADs anterior to the TPA to apply protraction force apically and through the center of resistance of the upper posterior teeth.

Then, protraction was started in the upper arch by using orthodontic separator elastics from the TADs to the hocks. So, placing hooks in the TPA about 10 mm apically to the bracket's slot level and placing two paramedian TADs anteriorly provide symmetrical bilateral movements. Therefore the upper posterior teeth could be protracted bodily without affecting upper and lower anterior teeth utilizing a power chain or elastic threads (Figure 10).

**FIGURE 11:**
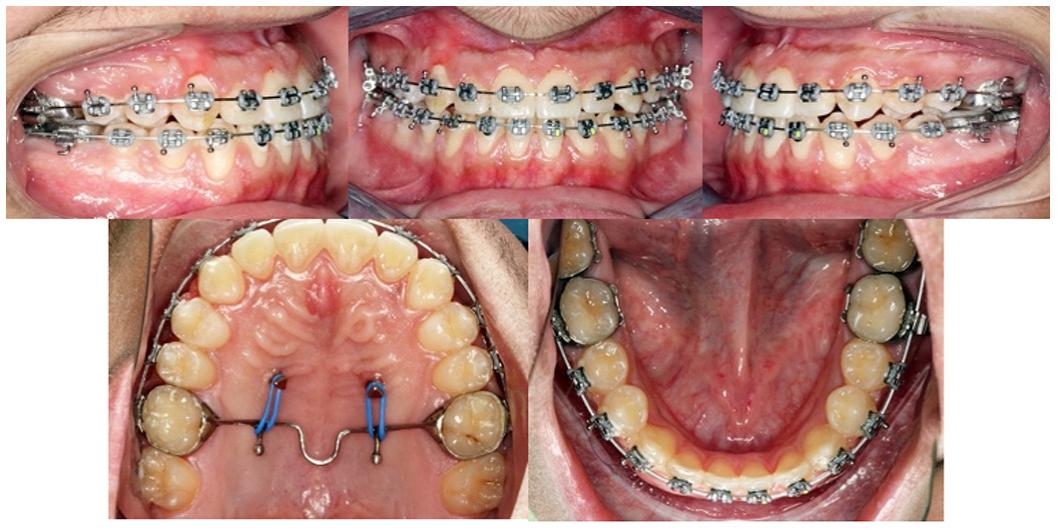
After all, spaces closed.

After all the spaces in the upper arch are closed with achieved Cl I canines and molars relationships on both sides, steel tied the TPA to the TADs. Then, box elastics were placed on both sides to enhance the occlusal settling. Progress panoramic and cephalometric radiographs were taken. Repositioning of some brackets were performed (Figure 11).

**FIGURE 12:**
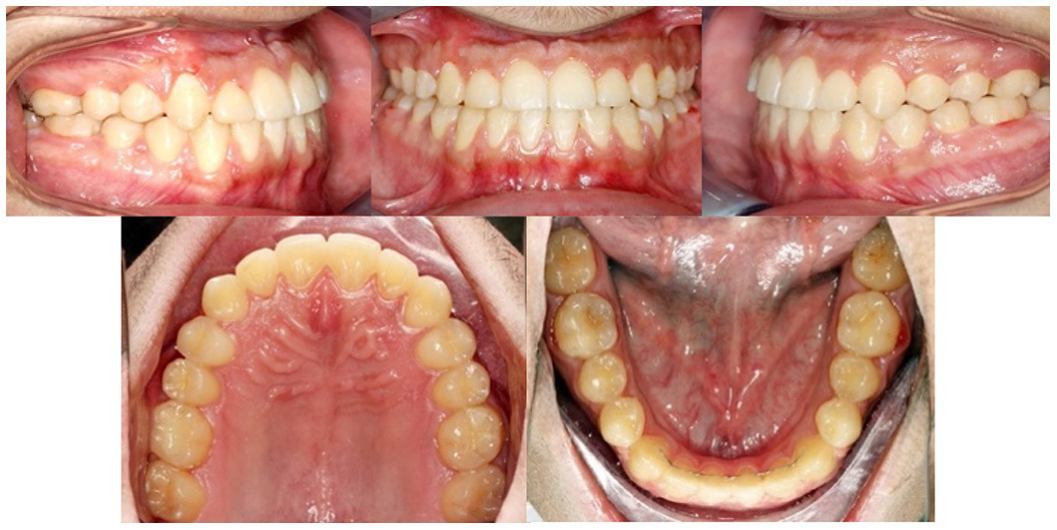
Post‐treatment intraoral photographs.

Finally, a lower fixed retainer was cemented, and the patient was debonded. Removable wraparound upper and lower retainers were delivered (Figure 12).

## 3 DISCUSSION

**FIGURE 13:**
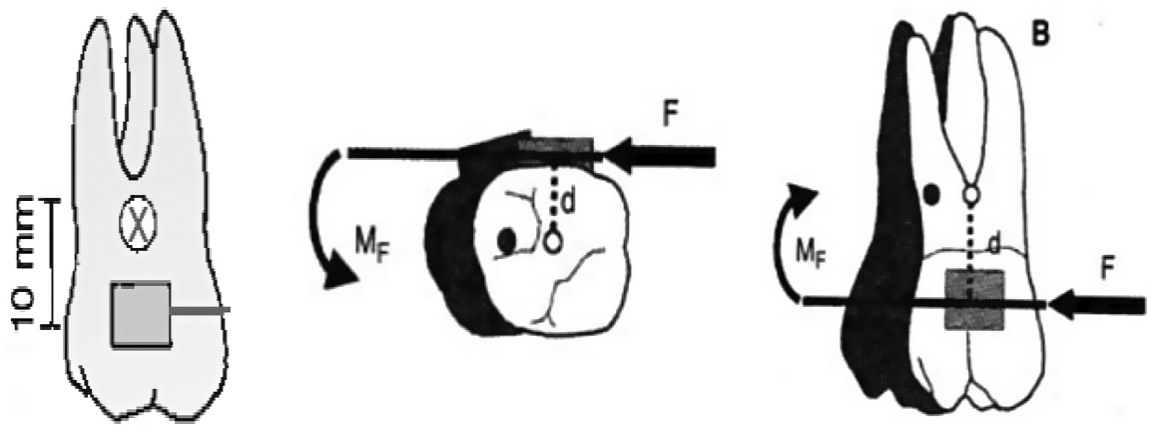
The force should be directed through the CR.

When an orthodontic approach cannot correct the malocclusion caused by a skeletal deformity completely, surgery will be the treatment of choice that will address the esthetics, functional stability, and treatment time objectives.^9^ In this case report, all these considerations were achieved with camouflage orthodontic treatment alone. Camouflage orthodontic treatment has been chosen over the surgical option in this patient due to the severity of skeletal discrepancy being within the moderate extent with acceptable soft tissue compensation. Although, by camouflage orthodontic treatment, the functional and esthetic needs could be addressed. Furthermore, the orthodontist should be aware of the factors that influence the orthodontic treatment planning for nonsurgical Class III malocclusion, including the patient's age, presence of functional shift, family history of malocclusion, direction, and pattern of the mandibular growth. The treatment was started with maxillary expansion to increase the arch perimeter and correct the intermolar width. Regarding this matter, some evidence indicates that the anterior part of the maxilla is severely deficient in patients with canine impactions.^10^ After the canine was retracted and leveled in its normal functional position with achieved positive overjet and overbite with Class I canine relationship on both sides, there was a ¼ unit Class III molar relationship with some spaces located anteriorly and the lower incisors inclination getting worse (Figure 9). It is well‐known that any force applied through the center of mass of a tooth will cause the entire tooth to move in the direction of the applied force, a process known in orthodontics as translation or bodily movement. (Figure 13).

**FIGURE 14:**
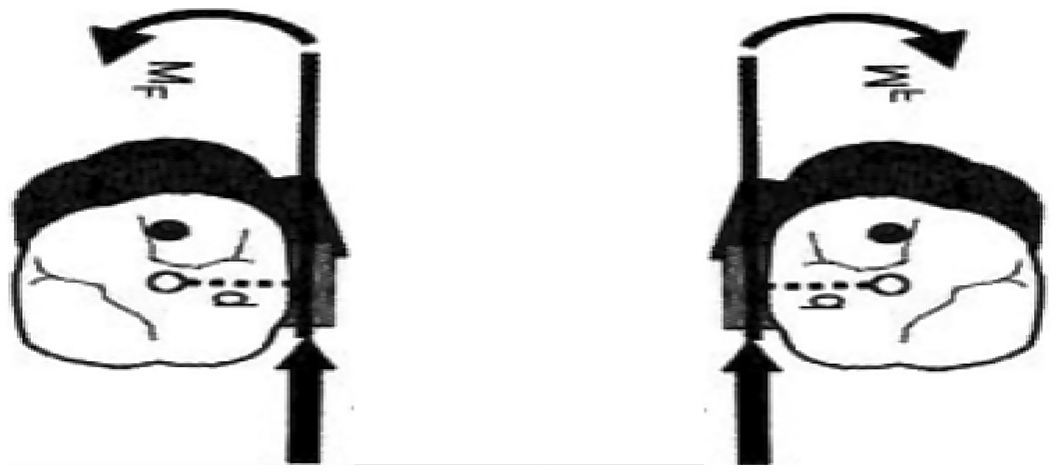
Palatally applied force to the first molars.

Although to move a tooth or teeth segment bodily, we should carefully evaluate some factors, including the amount and direction of the force, the relationship of the force applied to the center of resistance, generated movements in all dimensions, and how to control them. In most cases, the center of resistance of the maxillary first molars is about 8–10 mm apical to the bracket slot, and to move these teeth bodily, the force should be directed through the center of resistance to avoid any tipping movement and by using temporary anchorage devices. According to a new systematic review and meta‐analysis,^11^ orthodontic mini‐implants and direct anchorage with the ideal implant location in the palatal side versus the alveolar ridge can achieve maximum anchorage for en‐masse retraction. Further research is needed to determine whether direct anchorage is superior to indirect anchorage. But, even with palatally applied force, there will be a moment to rotate the upper molars mesially out (Figure 14). Placing hocks in the TPA about 10 mm apical to the bracket's slot level and placing two paramedian TADs anteriorly provide symmetrical bilateral movements.

The upper posterior teeth were protracted without affecting upper and lower anterior teeth using a power chain or elastic threads (Figure 10b). A tieback method of space closure with orthodontic separators was used, which has the more appropriate initial force and slower force decay, that will have a clinical value, approaching a lighter and more continued force.^12^

### 3.1 The treatment outcomes and critiques/limitations

#### 3.1.1 Skeletally

**FIGURE 15:**
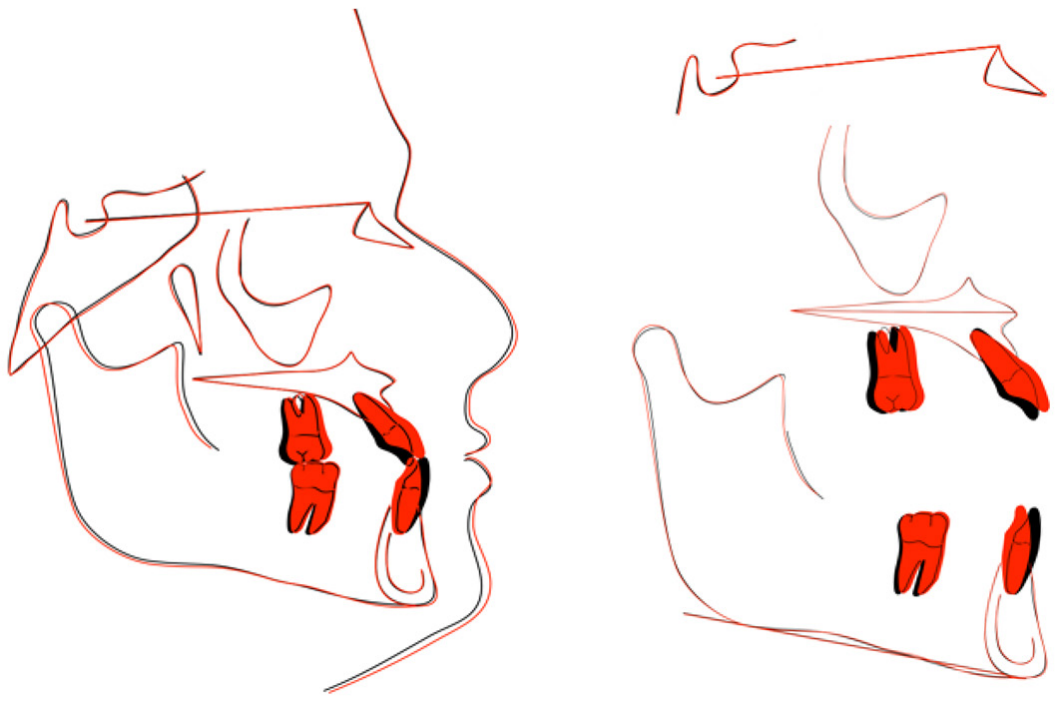
Superimposition of pre‐ and post‐treatment.

As presented in the part of the superimposition interpretation, no significant changes were seen in the patient profile and skeletal relationships. (This was the goal in the camouflage treatment) (Figure 15).

#### 3.1.2 Dentally

The inclination of the upper incisors was increased because of the camouflage treatment, while the lower incisors' proclination was within normal range. The overjet and overbite were good. All crowding and rotations were corrected. Class I molar and Class I canine relationships were achieved bilaterally. No significant changes were noted in the upper and lower lips positions due to the nonextraction and nonsurgical treatment approach.

#### 3.1.3 Psychologically

The patient reported that he was happy, especially after the correction of the anterior crossbite and improvement of his smile. In addition, he appreciated the positive change in the function.

The patient was presented initially with a mild‐to‐moderate severity of Class III malocclusion. The malocclusion was treated successfully by nonextraction, nonsurgical comprehensive orthodontic treatment and exposure, and retraction of the impacted tooth. The proposed treatment objectives were addressed efficiently with high patient satisfaction. To critique the treatment result for this patient, by starting with the treatment plan step: The ideal treatment plan for this case was the surgical approach and this treatment was advised to the patient from the beginning but he refused it and insisted on treating him without surgery. For nongrowing Class III skeletal malocclusion cases, the surgical option would provide the patient with excellent results and incredible changes in the soft tissue profile. As the treatment was going well with no complications, the patient insisted on debonding him as soon as possible as he had travel plans. So, debonding was done with the obtained results even though the patient could have benefited with a few more visits. For example, upper incisors could have benefited from slight extrusion to provide better retention of the result and improvement of the smile.

**FIGURE 16:**
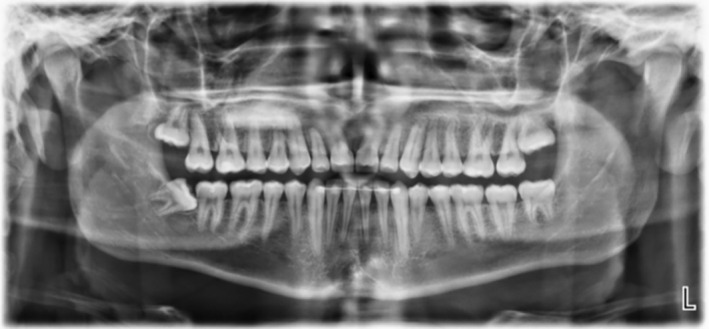
Post‐treatment panoramic radiograph.

Upper central incisors appeared on the post‐treatment panoramic radiograph (Figure 16) flared but they were acceptable clinically and if we see the preoperative panoramic radiograph, it is clear that the upper incisors roots were dilacerated and inconsistent with the angulation of the crowns. The patient would benefit from an upper 3 to 3 fixed retainer.

**FIGURE 17:**
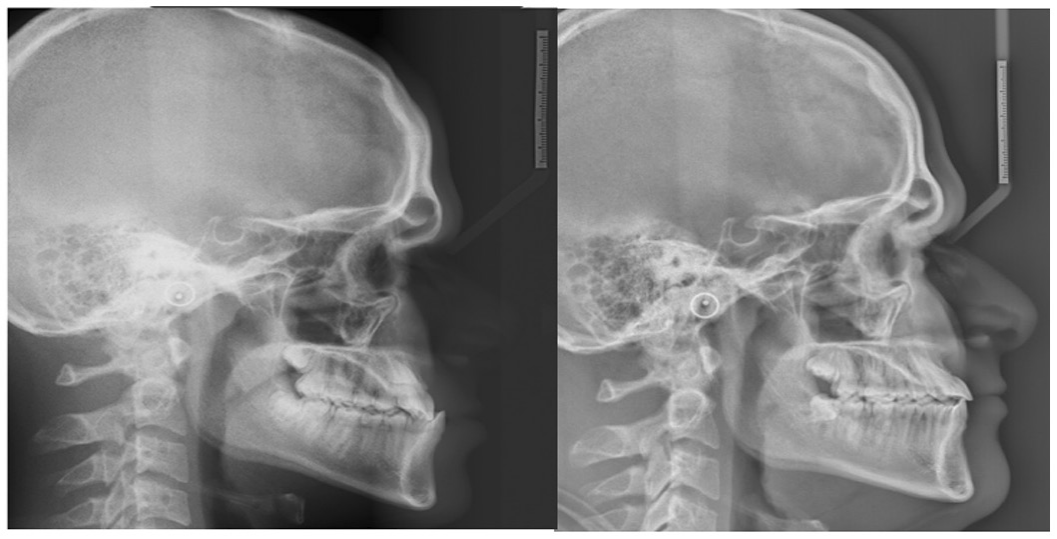
Pre and post‐treatment cephalometric radiographs.

The postoperative cephalometric radiograph is shown in (Figure 17).

### 3.2 Proposed retention strategy and rationale

**FIGURE 18:**
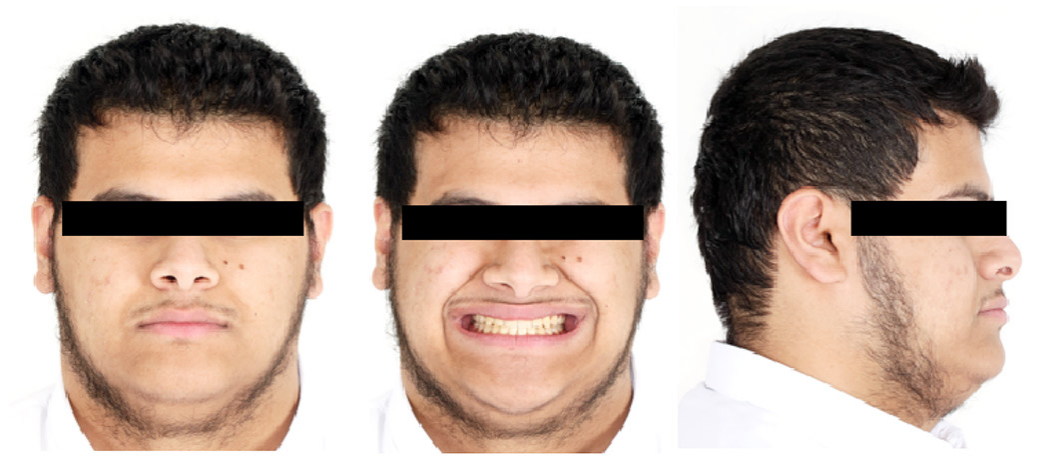
Post‐treatment extraoral photographs.

**FIGURE 19:**
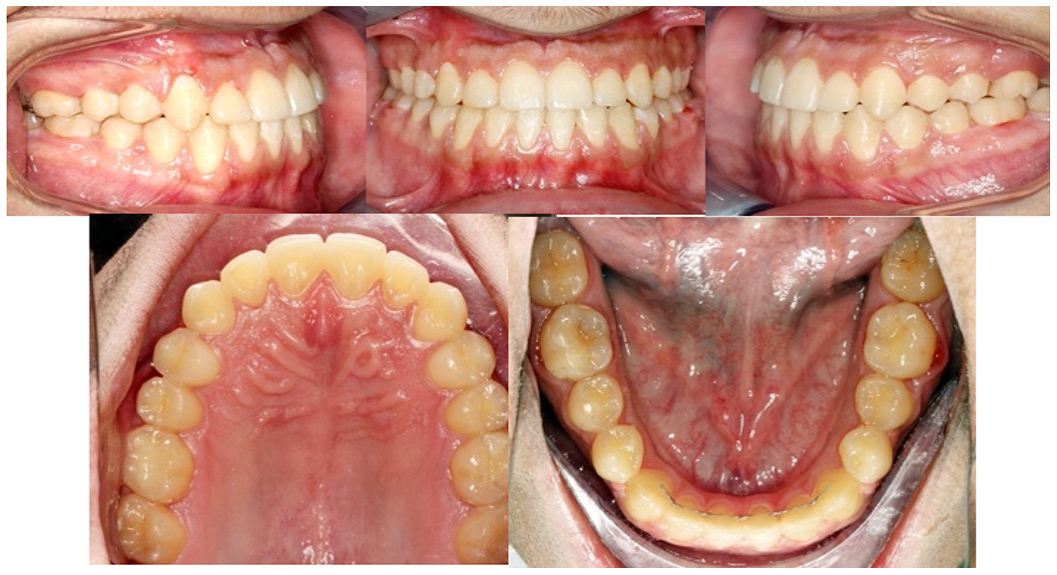
Post‐treatment intraoral photographs.

**FIGURE 20:**
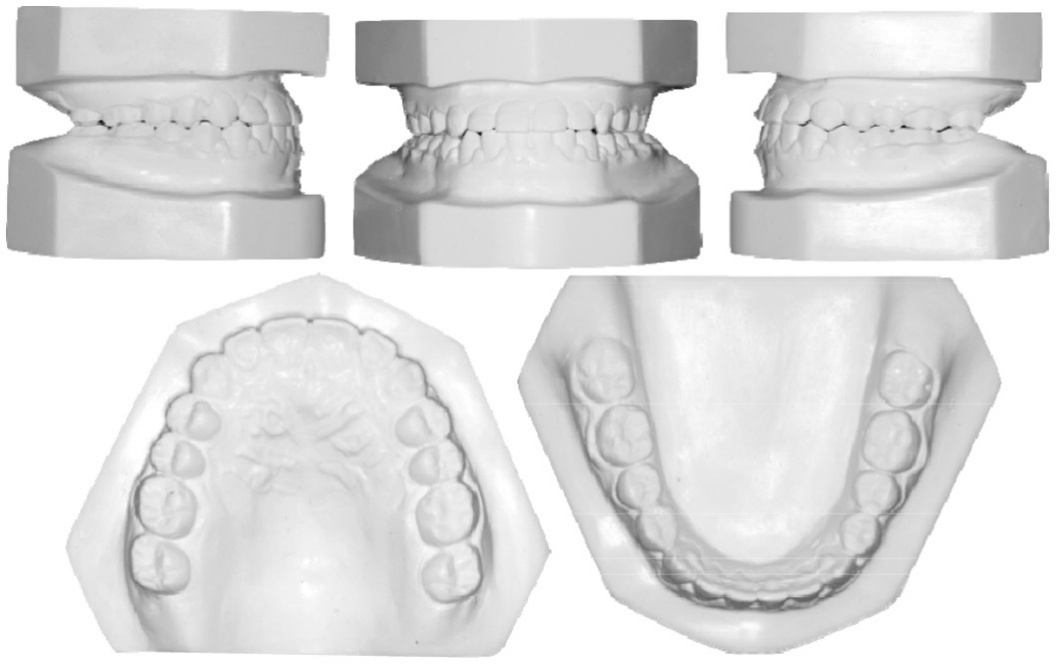
Post‐treatment dental casts.

An upper and lower removable wraparound Hawley retainers with lower 3–3 fixed retainers were used to prevent any rotations or crowding that usually developed by age as a result of reduced intercanine distance. Hawley retainers should be worn for a period of 6 months full time, followed by 6 months of nightwear, because that is associated with fewer relapses than 3 months full time and 3 months nights only.^13^ The final extraoral (Figure 18), intraoral (Figures 19), and cast photographs (Figure 20) are shown, respectively.

## 4 CONCLUSION

Maxillary teeth protraction via TADs placed in the palate showed to be an effective option in correcting Class III molar relationship with no required patient compliance compared to the protraction face mask or Class III elastics.

## AUTHOR CONTRIBUTIONS

**Saud Alotaibi:** Conceptualization; data curation; formal analysis; investigation; methodology; project administration; resources; software; supervision; validation; visualization; writing – original draft; writing – review and editing.

## CONFLICT OF INTEREST STATEMENT

The author declares that he has no competing interests.

## CONSENT

Signed informed consent was obtained from the patient for publication of this report and any related photographs.

## Data Availability

Not applicable.
